# Androgen and estrogen secretory dynamics in longitudinally followed moderately and late preterm infant girls

**DOI:** 10.1210/jendso/bvag036

**Published:** 2026-02-17

**Authors:** Carina Ankarberg-Lindgren, Kerstin Allvin, Jovanna Dahlgren

**Affiliations:** Department of Pediatrics, Gothenburg Pediatric Growth Research Center, Institute of Clinical Sciences, Sahlgrenska Academy, University of Gothenburg, Gothenburg 41685, Sweden; Department of Pediatrics, Gothenburg Pediatric Growth Research Center, Institute of Clinical Sciences, Sahlgrenska Academy, University of Gothenburg, Gothenburg 41685, Sweden; Department of Neonatology, Region Västra Götaland, Sahlgrenska University Hospital, Gothenburg 41685, Sweden; Department of Pediatrics, Gothenburg Pediatric Growth Research Center, Institute of Clinical Sciences, Sahlgrenska Academy, University of Gothenburg, Gothenburg 41685, Sweden; Department of Pediatric Endocrinology, Region Västra Götaland, Sahlgrenska University Hospital, Gothenburg 41685, Sweden

**Keywords:** mini-puberty, dihydrotestosterone, testosterone, estradiol, reference intervals, mass spectrometry

## Abstract

**Context:**

Mini-puberty is a period when the hypothalamic-pituitary-gonadal (HPG) axis is activated and is believed to create the basis for future fertility. In prematurely born females, delayed HPG axis activity and lower reproduction rates have been reported. However, the secretion dynamics of sex steroids in infant girls have not been fully elucidated,

**Objective:**

To study sex steroid secretory dynamics in girls born moderately to late preterm during infancy and to find out whether the degree of prematurity or low birth weight may lead to a disturbed sex steroid profile.

**Methods:**

From a population-based longitudinal cohort of 70 girls, born at gestational age 32.0 to 36.9 weeks at Sahlgrenska University Hospital, Gothenburg, Sweden, androgen and estrogen concentrations were analyzed during infancy with high-sensitivity mass spectrometry-based methods.

**Results:**

Serum androstenedione and estrone declined continuously during infancy. Testosterone and dihydrotestosterone (DHT) both showed surges around 1 to 2 months chronological age. The surges in estradiol varied individually, with the peak level occurring either before 2 months or at 2 to 3 or 5 to 7 months. At 5 months corrected age, estradiol correlated inversely to gestational age (r = −0.47, *P* = .000). At 10 months corrected age, gestational age correlated with androstenedione (r = 0.44, *P* = .002), testosterone (r = 0.32, *P* = .031), DHT (r = 0.31, *P* = .043), and estrone (r = 0.43, *P* = .004).

**Conclusion:**

In girls born moderately to late preterm, DHT, estradiol, and testosterone show a postnatal activity with synchronized surges for DHT and testosterone, while estradiol is secreted with an individual dynamic. Androstenedione and estrone show no surge during infancy. Degree of prematurity but not birth weight influences sex steroid secretion.

The endocrine system plays a crucial role in regulating various physiological processes through the secretion of hormones, and its proper function is essential for the normal growth and development of infants. After birth, the newborn loses connection to the endocrinologically active placenta, and hormones such as estrogens and progesterone decrease in the neonate's circulation [1]. During infancy, rapid endocrine changes take place. The activation of the hypothalamic-pituitary-gonadal (HPG) axis in the first months of life was first described in the 1970s, when extraction radioimmunoassays enabled the determination of serum sex hormone levels [2, 3]. The changes are not only due to disruption of the active placenta, but we know now that the infant has active gonads. This postnatal activation, referred to as “mini-puberty,” is a unique window to diagnose HPG axis disorders. It is believed that mini-puberty is crucial for the development of the genital organs and creates the basis for future fertility. In boys, testicular growth and increased penile size are evident, while the physiological role of mini-puberty in girls is still not well understood [4]. Nevertheless, girls exhibit ovarian follicular development [5, 6], and effects on later mammary size and uterine growth have been documented [7, 8].

Previous studies have shown that girls born small for gestational age (SGA) or with low birth weight (BW) tend to have an earlier onset of puberty and reduced uterine and ovarian size than females of normal BW [9, 10]. Furthermore, there are indications for delayed HPG axis activity in infant girls born preterm and lower reproduction rates in prematurely born adults [5, 11]. Further studies are necessary to understand the associations between altered mini-puberty and prematurity and low BW. Due to the low concentrations found in infant children and the small sample volumes, sex steroid determination is an analytical challenge, requiring highly sensitive and specific analytical assays, such as tandem mass spectrometry (MS/MS)-based methods [12, 13].

Gonadotrophin levels are high during the first 3 months of life but decrease toward the age of 6 months, except for FSH levels in girls, which remain elevated until 3 to 4 years of age [5, 14]. After this, the HPG axis remains quiescent until the onset of puberty. The postnatal gonadotrophin surge results in gonadal activation in both sexes. In male infants, testosterone levels are low in cord blood, start to increase after 1 week of age, peak to pubertal levels between 1 and 3 months after birth, and decline to prepubertal levels by about 6 months of age [15, 16]. In female infants, in contrast, little is known about the androgen secretory dynamics. Estrogen levels are high in cord blood of both sexes and decrease rapidly during the first postnatal days [3]. Unlike testosterone in male infants, estradiol concentrations are presumed to fluctuate or have a biphasic pattern in female infants [7, 17]—presumed to reflect the cyclic nature of maturation/atrophy of ovarian follicles. However, with limited and inconclusive data from longitudinal cohorts of infant girls, the dynamics of sex steroids in the individual infant girl have not been fully elucidated, and a thorough evaluation is needed. The major challenge is getting parents of full-term infants to agree to repeated examinations and blood sampling; we therefore aimed to study healthy moderately to late preterm infants.

In this longitudinal study we quantified androstenedione, testosterone, dihydrotestosterone (DHT), estradiol, and estrone in infant girls born moderately to late preterm using high-sensitivity MS/MS-based methods. We aimed to evaluate the individual dynamics of androgens and estrogens during first months of life. Furthermore, we aimed to evaluate whether there is an association between changes in sex steroids during infancy and gestational age (GA) or size at birth.

## Materials and methods

### Subjects

The study population was recruited prospectively as a population-based cohort at the 2 delivery wards at Sahlgrenska University Hospital in Gothenburg, Sweden. Two hundred forty-seven neonates (110 girls, 137 boys) born between September 2002 and June 2004 were included in the cohort. The present study is a subgroup of the larger cohort, for whom sex steroid data in boys have already been published [16, 18]. Of the 110 girls, 82 girls were singletons and 28 were twins. Girls with serious medical conditions, malformations, or chromosomal anomalies were excluded. Seventy singleton girls were longitudinally followed with blood samplings and included in this study. Fifteen girls were born at GA 32.0 to 33.9 weeks, classified as moderately preterm, and 55 girls were born at GA 34.0 to 36.9 weeks, classified as late preterm. In all infants, the estimated date of delivery (EDD) had been determined by ultrasonography performed at gestational week 16 to 18.

All 70 infants followed the same examination schedule. Because all girls were born moderately to late preterm, the time points at which measurements were taken were corrected according to EDD. Auxological measurements were registered at birth; at EDD; and at 2, 5, and 10 months thereafter, in the following text referred to as 0, 2, 5, and 10 months corrected age, respectively. Weight was measured using digital infant scales. Length was measured with the infant in a supine position on an electronic infant-length board. Thirty-one girls were born with BW less than 2500 g, which is the definition for low BW by the World Health Organization [19]. Thirteen out of the 70 girls were born SGA, defined as BW or birth length below −2 SD score (SDS), and of these, 9 were born severely SGA, defined as BW and birth length below −3 SDS according to the Swedish reference for newborns [20].

In order to further understand expected hormone levels in girls during their first year of life, and to provide, as far as possible, age-specific reference intervals, a subset of girls born before GA 34.0 and girls with severe SGA were excluded. In total, 50 girls were included for estimation of age-specific reference intervals. Their GA ranged from 34.0 to 36.7 weeks with an average of 36.0 weeks. Median BW in grams was 2698 (range 1745-3815), median BW SDS −0.2 (range −2.5-+2.4), and median birth length SDS +0.1 (range −2.4-+2.8).

### Blood sampling

Umbilical venous blood was collected directly after birth in 62 girls. These are a subgroup of a larger cohort, for whom sex steroid data in umbilical venous blood have already been published [18]. Venous blood samples were collected once a week if the girl was admitted to a neonatal ward after birth. Blood was also collected 3 days after birth (postnatal screening); around EDD; and at 2, 5, and 10 months thereafter. The median number of blood samples per infant girl was 4 and ranged from 2 to 7.

### Sex steroid determinations

Serum androstenedione, testosterone, DHT, estrone, and estradiol concentrations were simultaneously determined by gas chromatography-MS/MS, as previously described [21]. The lower limit of detection (LLOD) was 0.10 nmol/L for androstenedione, 0.10 nmol/L for testosterone, 0.03 nmol/L for DHT, 9 pmol/L for estrone, and 2 pmol/L for estradiol. Total coefficient of variation (CV) for androstenedione was 17% at 0.5 nmol/L and 13% at 2 nmol/L; for testosterone, total CV was 16% at 0.3 nmol/L and <10% at >1.5 nmol/L; for DHT, it was 15% at 0.06 nmol/L and 10% at 0.20 nmol/L; for estrone it was 16% at 21 pmol/L and 11% at ≥ 100 pmol/L; and for estradiol it was 19% at 8 pmol/L and 6% at ≥ 36 pmol/L. The imprecision data were calculated on pediatric samples, yielding the relatively high CV as it approaches the LLOD. In 20 out of 70 samples from moderately premature girls and in 67 out of 218 samples from late preterm girls, the sera volumes were not large enough for the ordinary protocol of 200 µL. Instead, volumes of 100 to 175 µL were used and the LLOD adjusted accordingly. The reduced sample volumes and relatively high CV caused uncertainty in the low ranges close to or below the LLOD but did not affect results at higher levels.

### Statistical analysis

Hormone data is presented as median (2.5-97.5 percentiles). In calculations, hormone concentrations below the LLOD were assigned the value LLOD/2. The Welch T-test for independent samples was used for comparisons between groups. Correlation analyses were performed using Pearson correlations. When nonlinear, they were performed on logarithmic values. The software IBM SPSS Statistics for Windows, version 29.0 (IBM Corp., Armonk, NY, USA) was used for statistical analyses. Figures were drawn using OriginLab 2021 (Northampton, MA, USA).

### Ethical considerations

The study was approved by the Ethics Committee of the Medical Faculty of the University of Gothenburg (approval number Ö-562-01). Informed consent was obtained from the participants' parents.

## Results

### BW

No correlation was found between BW and sex steroid concentrations. However, BW SDS correlated with androstenedione at 0 months corrected age (r = 0.40, *P* = .003) and with DHT at 10 months corrected age (r = 0.35, *P* = .024).

### GA

**Figure 1:**
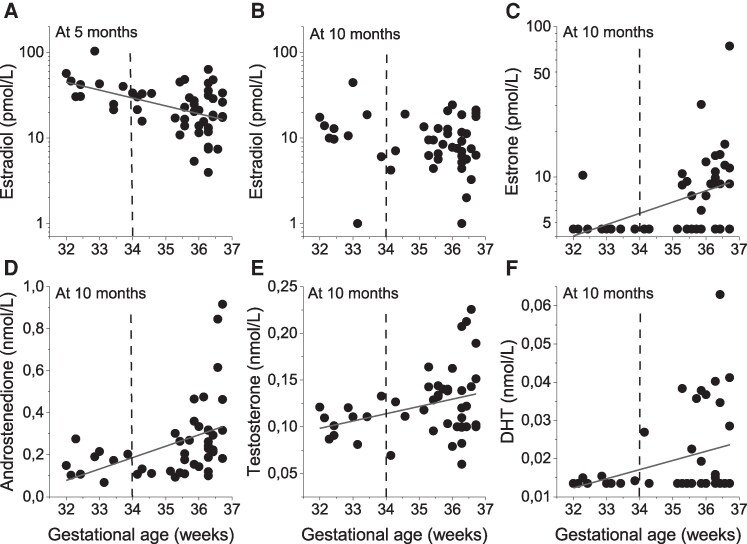
Sex steroids in relation to gestational age in 70 preterm infant girls. Correlations for (A) estradiol concentrations at 5 months corrected age (r = −0.47, *P* = .000) and (B) estradiol (r = −0.16, *P* = .309), (C) estrone (r = 0.43, *P* = .004), (D) androstenedione (r = 0.44, *P* = .002, (E) testosterone (r = 0.32, *P* = .031), and (F) DHT (r = 0.31, *P* = .043) concentrations at 10 months corrected age. Abbreviations: DHT, dihydrotestosterone.

No correlation was found between GA and sex steroid concentrations at 0 months or 2 months corrected age. However, at 5 months corrected age, GA correlated inversely with estradiol; girls born before GA 34.0 weeks had significantly higher estradiol concentrations at 5 months corrected age compared to those with GA >34.0 weeks (*P* = .022) (Fig. 1A). In contrast, girls born before GA 34.0 weeks had significantly lower estrone concentrations at 5 months corrected age compared to those with GA >34.0 weeks (*P* = .041), but no significant correlation was found (r = 0.12, *P* = .378, data not shown).

At 10 months corrected age (Fig. 1B-1F), GA showed no relationship with estradiol but correlated with estrone, androstenedione, testosterone, and DHT; girls born before GA 34.0 had significantly lower estrone (*P* = .022), androstenedione (*P* = .005), testosterone (*P* = .006), and DHT (*P* = .001) but not estradiol (*P* = .268).

### Androgens

**Figure 2:**
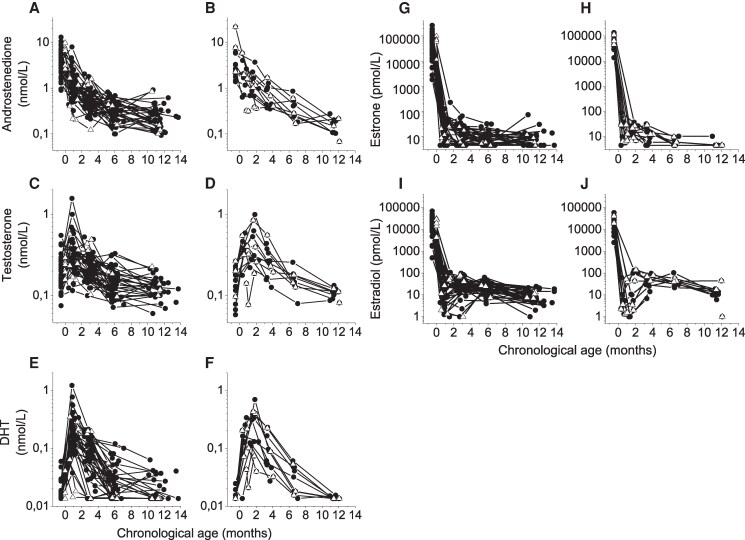
Individual serum concentrations for (A, B) androstenedione, (C, D) testosterone, (E, F) DHT, (G, H) estrone, and (I, J) estradiol in longitudinally followed preterm infant girls. The left panels show 55 infant girls born late preterm, and the right panels show 15 infant girls born moderately preterm. The first sampling presents hormone levels in cord blood, whereas the other samples show hormone concentrations in serum. Open triangles depict girls born with birth weight below −3 SD score. Abbreviations: DHT, dihydrotestosterone.

**Figure 3:**
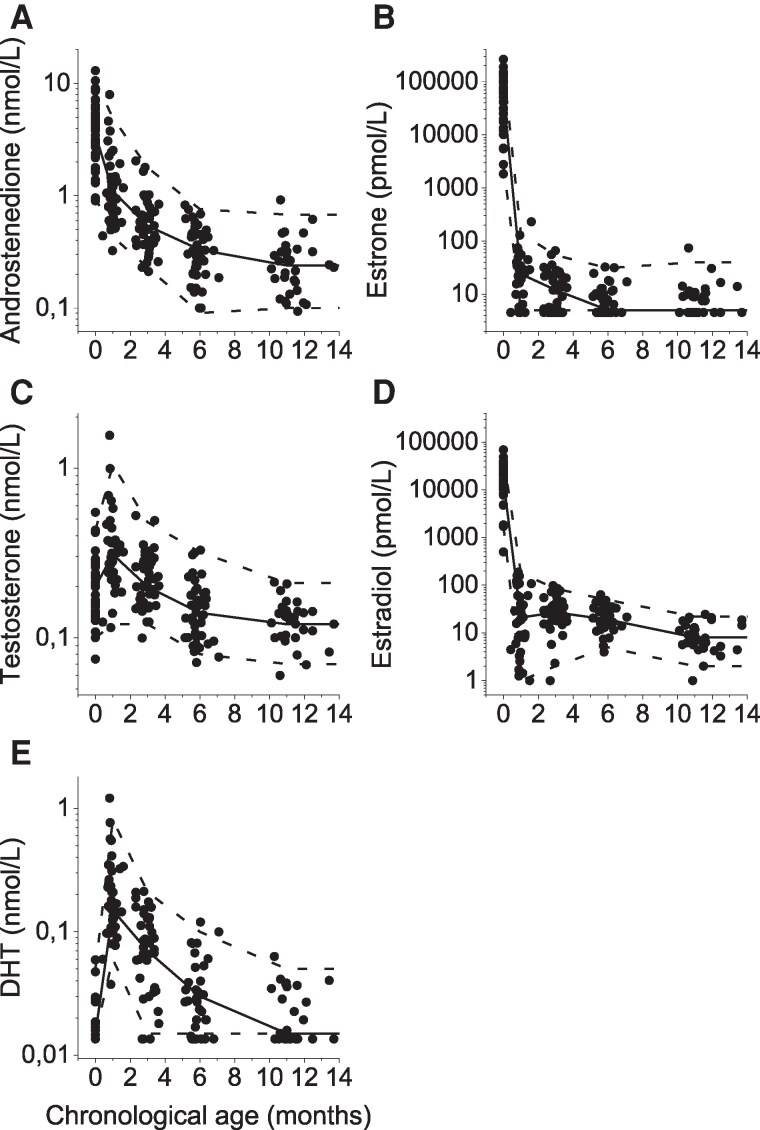
Serum concentrations of (A) androstenedione, (B) estrone, (C) testosterone, (D) estradiol, and (E) DHT in relation to chronological age in 50 healthy late preterm infant girls. The first sampling presents hormone levels in cord blood, whereas the other samples show hormone concentrations in serum. The solid line depicts median value and the dashed lines 2.5 and 97.5 percentiles. Abbreviations: DHT, dihydrotestosterone.

Serum androstenedione showed a synchronized pattern, with the highest levels in cord blood. Androstenedione concentrations thereafter declined continuously from 0 months to 10 months corrected age (Figs. 2A, 2B, and 3A and Table 1).

**Table 1:** Androgen and estrogen concentrations at birth (cord blood) and at 0, 2, 5, and 10 months' corrected age (serum) in 50 longitudinally followed late preterm infant girls

Corrected age (months)	Chronological age (months)	n	Androstenedione (nmol/L)	Testosterone (nmol/L)	DHT (nmol/L)	Estrone (pmol/L)	Estradiol (pmol/L)
At birth (cord blood)	At birth (cord blood)	43	3.5 (0.97-10.5)	0.20 (0.10-0.43)	<0.03 (<0.03-0.05)	58 601 (1836-264 322)	15 149 (1711-48 005)
0	1.0 (0.3-1.6)	37	1.1 (0.43-5.0)	0.31 (0.12-1.1)	0.15 (0.06-0.81)	24 (<9-141)	21 (<2-182)
2	3.0 (2.3-3.6)	43	0.54 (0.23-1.8)	0.20 (0.12-0.48)	0.07 (<0.03-0.21)	12 (<9-54)	27 (2-84)
5	5.8 (5.2-7.1)	40	0.33 (0.09-0.76)	0.14 (0.08-0.32)	0.03 (<0.03-0.10)	<9 (<9-31)	19 (5-49)
10	11.0 (10.1-13.7)	33	0.24 (0.10-0.68)	0.12 (0.07-0.21)	<0.03 (<0.03-0.05)	<9 (<9-40)	8 (<2-22)

Chronological age is presented as median with range in parentheses. Hormone concentrations are presented as median with 2.5 to 97.5 percentiles in parentheses.

Abbreviation: DHT, dihydrotestosterone.

Serum testosterone showed a synchronized pattern with a surge at 1 month chronological age for those born late preterm (Figs. 2C and 3) and at 2 months chronological age for those born moderately preterm (Fig. 2D), which for both corresponds to 0 months corrected age. Thereafter testosterone levels declined to the lowest level at around 1 year of age (Table 1 and Fig. 3).

Serum DHT followed the same pattern as testosterone, with a synchronized surge at 1 month chronological age for those born late preterm (Figs. 2E and 3) and at 2 months chronological age for these born moderately preterm (Figs. 2F). Thereafter, DHT declined to the same level as in cord blood toward 1 year of age (Table 1 and Fig. 3).

### Estrogens

Estrone concentrations showed a dramatic drop from 58 601 (1836-264 322) pmol/L in cord blood to 24 (<9-141) pmol/L at 1 month chronological age (ie, 0 months corrected age). Thereafter, a continuous decline in serum was observed, with most girls reaching a baseline level at around 6 months chronological age (Table 1, Figs. 2G, 2H, and 3B).

**Figure 4:**
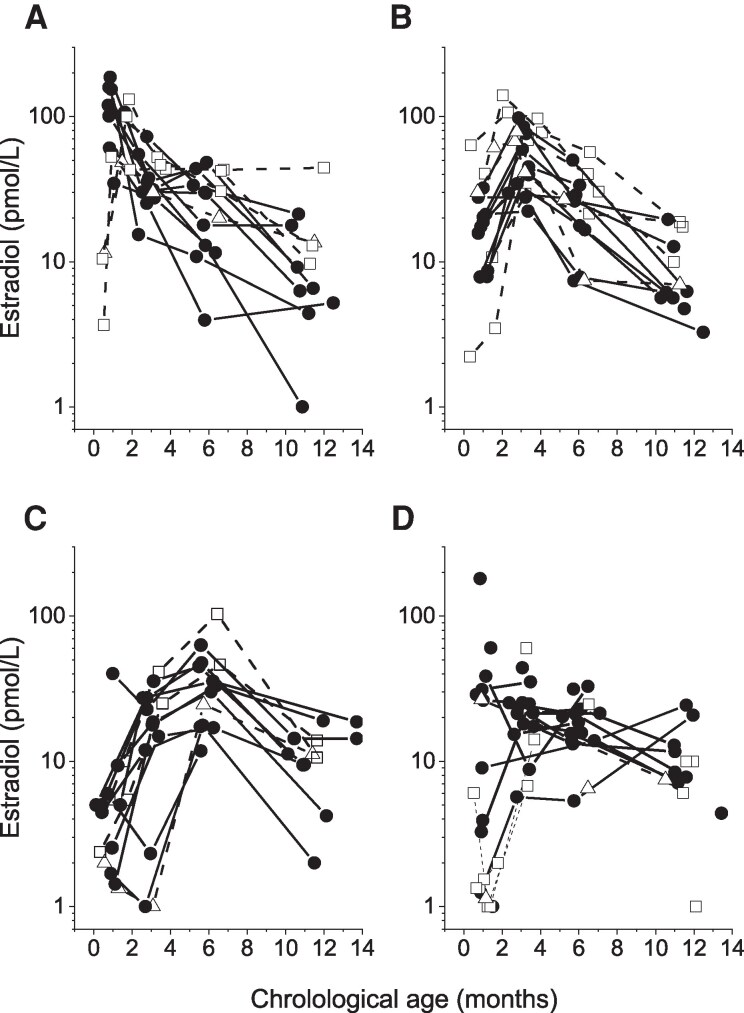
Serum estradiol concentrations in 70 longitudinally followed infant girls born at gestation age 32.0 to 36.9 weeks. Secretion pattern with peak levels (A) before 2 months of age, (B) at 2 to 3 months, and (C) at 5 to 7 months of age. Panel (D) shows 2 girls who had their highest estradiol concentration at 12 months of age and 25 profiles where the samplings were not fully longitudinal (fewer than 4) and therefore the peak value was unclear. Open symbols with dashed lines represent estradiol secretion patterns in girls born before gestational age 34.0 weeks (squares) and girls with birth weight below −3SD score (triangles).

Although all individual estradiol profiles showed a dramatic drop from cord blood levels to sampling at 1.0 (0.3-1.6) months chronological age, no uniform secretory dynamics in serum estradiol were distinguished (Fig. 2I and 2J). The individual surge occurred at different ages in these longitudinally followed girls. Fourteen girls had their peak estradiol level of 104 (35-186) pmol/L at 0.9 (0.8-1.8) months chronological age, which was 0 months corrected age (Fig. 4A); 16 girls had their peak of 59 (22-140) pmol/L at 3.1 (2.0-3.4) months (Fig. 4B); 13 girls had their peak with a concentration of 33 (12-103) pmol/L at 5.7 (5.5-6.6) months (Fig. 4C), and 2 girls had their peak value of 23 (21-24) pmol/L at 11.8 (11.6-12.0) months (Fig. 4D). In the remaining 25 girls, sampling was not fully longitudinal (fewer than 4 venous blood samples), and crucial sampling times were missing to distinguish any peak or secretion dynamics (Fig. 4D). As shown in the previous section, girls born before GA 34.0 weeks had significantly higher estradiol around 6 months compared to those with GA > 34.0 weeks. Although the higher estradiol levels are visually distinguished in Fig. 4A to 4C (open square symbols), the various times for peak levels were not related to either GA or to birth size. Age-specific reference intervals for girls born late premature are shown in Table 1 and Fig. 3.

## Discussion

In the present longitudinal study, using a high-sensitivity mass spectrometry-based method, we were able to demonstrate a postnatal activity of testosterone, DHT, and estradiol in moderately to late preterm infant girls. The degree of prematurity seems to affect the steroid synthesis in infant girls.

Unlike for boys in the same cohort [16], BW did not impair sex steroid patterns in girls. Only BW SDS correlated with androstenedione at 0 months corrected age and with DHT at 10 months corrected age. In this study, girls born moderately preterm had significantly higher estradiol concentrations at 5 months corrected age compared to those born late preterm, even though they were chronologically older than their counterparts. Despite the small number of samples in the present study, the results here probably reflect an enhanced activity of the HPG axis in moderately preterm girls, like that seen in premature boys with higher testosterone levels [5, 22]. On the other hand, girls born moderately preterm had significantly lower androgen concentrations at 10 months corrected age, which may be explained by their more advanced chronological age, with decreasing levels toward the age of 1 year.

After full-term parturition, involution of the neonate's adrenals occurs, with a drop in adrenal hormones such as androstenedione, dehydroepiandrosterone sulfate, and cortisol [23]. However, according to previous research in preterm babies, the activity in the adrenal fetal zone continues to EDD and then diminishes [24]. In contrast, our study of moderately to late preterm infant girls demonstrated a synchronized pattern, and no delay in androstenedione decrement was observed.

To our knowledge, this is the first longitudinal study of androstenedione, testosterone, and DHT in girls during the first year of life performed with a high-sensitivity MS/MS based method. Previous studies, performed with immunoassays, which are known to lack sensitivity and accuracy to determine testosterone in cord blood and in neonates [12, 25], have indicated that girls, at birth, have serum testosterone concentrations similar to those in adult females and experience a rapid fall in testosterone concentrations during the first 2 months after birth [26, 27]. The present study resulted in new insights. Testosterone concentrations in the newborn girls in our study were lower compared to previous studies—about half the level in adult women [28]. Concordant testosterone levels have also been established at 2 weeks and 4 weeks old in healthy neonates as well as in preterm girls [29-31]. The levels of androstenedione in neonate girls, on the other hand, were comparable to levels in adult women [28], and corresponding androstenedione concentrations have also been shown in healthy neonates at 2 weeks old [29]. The novelty of this study is that both testosterone and DHT showed a synchronized secretion dynamic with a surge at 1 month for girls born late preterm and at 2 months for girls born moderately preterm. Hence, the surge occurred at about the same age when corrected for EDD. However, in a population with girls born at term, this peak might be difficult to distinguish as the postnatal surge may then appear closer to birth. In a previous longitudinal study of boys originating from the same cohort, we found a synchronized surge in testosterone and DHT at around 3 months chronological age [16]. In the current study, we found that the postnatal surge in testosterone and DHT is earlier in girls and has less magnitude than in boys. In girls, we presume the postnatal surge to be of ovarian origin and thus to reflect mini-puberty.

The Copenhagen mini-puberty study previously reported a biphasic pattern of estrone and estradiol during mini-puberty, with the first peak timed around 15 to 27 days and the second peak around 3.5 to 4 months [17]. In our study, with samples taken longitudinally, we cannot confirm a clear biphasic estrogen pattern in girls in the first year of life. No synchronized dynamic in estrone secretion was observed in the present study—only a constant decrease until 6 months of age. This is not surprising, as estrone is synthesized from androstenedione, which in turn is derived from the adrenals and therefore presents the same secretion pattern. Nevertheless, in our longitudinal study, we found that girls may have their estradiol peak at different time points during the first year, either before 2 months or between 2 and 3 months or even between 5 and 7 months. The levels were generally higher than those found in boys during infancy [16]. It is known that both sexes have estrogen-responsive tissue during infancy, and the higher estradiol levels in girls may explain why girls are more frequently shown to have larger breast tissue size compared to boys during infancy [32].

The strength of our study is the longitudinal design with regular serial blood sampling in a period when rapid developmental changes occur and the use of a high-sensitivity MS/MS-based method. Limitations of the study are the lack of infant girls born at term, the relatively small population, the lack of gonadotrophin determinations, and a sampling frequency that was inadequate for evaluation of estradiol secretion pattern in 25 out of 70 samples.

In conclusion, this report provides new insights into the postnatal secretion dynamics of androgens and estrogens in girls. We show that girls born moderately to late preterm exhibit synchronized surges in testosterone and DHT around 1 to 2 months of age, which corresponds to their EDD. Serum estradiol concentrations in infant girls peak individually, regardless of GA and birth size. At around 6 months of age, estradiol levels are higher in moderate preterm infant girls compared to those born late preterm, most likely due to an increased postnatal HPG axis activation. We also show that the adrenal-derived androstenedione and estrone concentrations decline continuously during the first year. Whether our results are also applicable to full-term girls merits further study.

## Data Availability

Restrictions apply to the availability of some or all data generated or analyzed during this study to preserve patient confidentiality or because they were used under license. The corresponding author will on request detail the restrictions and any conditions under which access to some of the data may be provided.

## Abbreviations

BW: birth weight
CV: coefficient of variation
DHT: dihydrotestosterone
EDD: estimated date of delivery
GA: gestational age
HPG: hypothalamic-pituitary-gonadal
LLOD: lower limit of detection
MS/MS: tandem mass spectrometry
SDS: SD score
SGA: small for gestational age
